# Genetic Characterization of Multiple Components Contributing to Fusarium Head Blight Resistance of FL62R1, a Canadian Bread Wheat Developed Using Systemic Breeding

**DOI:** 10.3389/fpls.2020.580833

**Published:** 2020-10-26

**Authors:** Wentao Zhang, Kerry Boyle, Anita L. Brûlé-Babel, George Fedak, Peng Gao, Zeinab Robleh Djama, Brittany Polley, Richard D. Cuthbert, Harpinder S. Randhawa, Fengying Jiang, François Eudes, Pierre R. Fobert

**Affiliations:** ^1^Aquatic and Crop Resources Development, National Research Council of Canada, Saskatoon, SK, Canada; ^2^Department of Plant Science, University of Manitoba, Winnipeg, MB, Canada; ^3^Ottawa Research and Development Centre, Agriculture and Agri-Food Canada, Ottawa, ON, Canada; ^4^Aquatic and Crop Resources Development, National Research Council of Canada, Ottawa, ON, Canada; ^5^Swift Current Research and Development Centre, Agriculture and Agri-Food Canada, Swift Current, SK, Canada; ^6^Lethbridge Research and Development Centre, Agriculture and Agri-Food Canada, Lethbridge, AB, Canada

**Keywords:** Fusarium head blight, resistance components, QTL, genetic architecture, DON, SNP, flowering

## Abstract

Fusarium head blight (FHB) is a devastating fungal disease of small-grain cereals that results in severe yield and quality losses. FHB resistance is controlled by resistance components including incidence, field severity, visual rating index, Fusarium damaged kernels (FDKs), and the accumulation of the mycotoxin deoxynivalenol (DON). Resistance conferred by each of these components is partial and must be combined to achieve resistance sufficient to protect wheat from yield losses. In this study, two biparental mapping populations were analyzed in Canadian FHB nurseries and quantitative trait loci (QTL) mapped for the traits listed above. Nine genomic loci, on 2AS, 2BS, 3BS, 4AS, 4AL, 4BS, 5AS, 5AL, and 5BL, were enriched for the majority of the QTL controlling FHB resistance. The previously validated FHB resistance QTL on 3BS and 5AS affected resistance to severity, FDK, and DON in these populations. The remaining seven genomic loci colocalize with flowering time and/or plant height QTL. The QTL on 4B was a major contributor to all field resistance traits and plant height in the field. QTL on 4AL showed contrasting effects for FHB resistance between Eastern and Western Canada, indicating a local adapted resistance to FHB. In addition, we also found that the 2AS QTL contributed a major effect for DON, and the 2BS for FDK, while the 5AL conferred mainly effect for both FDK/DON. Results presented here provide insight into the genetic architecture underlying these resistant components and insight into how FHB resistance in wheat is controlled by a complex network of interactions between genes controlling flowering time, plant height, local adaption, and FHB resistance components.

## Introduction

Fusarium head blight (FHB), or scab, mainly caused by the fungus *Fusarium graminearum* Schwabe [telomorph: *Gibberella zeae* Schw. (Petch)], is the most serious fungal disease affecting bread and durum wheat production in Canada (Gilbert and Tekauz, 2000). Fusarium damaged kernels (FDKs) are typically shriveled (or shrunken) and white or pink in appearance (Gilbert and Tekauz, 2000; Bai and Shaner, 2004). The lighter weight of FDKs and the high level of FDKs within a commercial wheat crop result in severe yield and quality losses (Gilbert and Tekauz, 2000; McMullen et al., 2012). FHB is also a food and feed safety concern due to the contamination of grain by the mycotoxin deoxynivalenol (DON) (Gilbert and Tekauz, 2000). The disease has become a more serious threat to wheat production for farmers with trends toward Fusarium isolates that produce higher levels of DON and more frequent FHB outbreaks across the Western Canadian Prairies (Gilbert and Tekauz, 2000). The warm and moist weather conditions experienced in 2014 and 2016, which favor Fusarium infection, caused huge losses for Saskatchewan farmers, with >50% of seed samples infected with Fusarium^1^. Annual losses attributed to FHB in Canada are in the hundreds of millions of dollars (Haile et al., 2019).

Chemical fungicides and agronomic practices have proven to be only partially effective at controlling FHB (Gilbert and Tekauz, 2000; McMullen et al., 2012), with genetic resistance offering the most effective approach for limiting the economic and ecological impacts of the disease (Gilbert and Tekauz, 2000; Bai and Shaner, 2004; McMullen et al., 2012; Gilbert and Haber, 2013). The genetics of FHB resistance is complex and contains multiple components. The resistance to the initial plant infection measured by the incidence of infection in the presence of natural or augmented inoculum (e.g., spray inoculation) is referred to as type I resistance (Mesterhazy, 1995; Mesterhazy et al., 1999; Bai and Shaner, 2004). Resistance to fungal spread across the wheat head and measured by the severity of infection is referred to as type II resistance (Mesterhazy, 1995; Mesterhazy et al., 1999; Bai and Shaner, 2004). Wheat can also display different levels of resistance to kernel infection, tolerance to infection, and accumulation of DON toxin (Miller et al., 1985; Mesterhazy, 1995; Mesterhazy et al., 1999; Bai and Shaner, 2004). Previous studies indicate that the genetic architecture of these different components are either only partially shared, or independent (Mesterhazy, 1995; Steiner et al., 2004; Lv et al., 2014). Therefore, it can be hypothesized that, in general, combining different types of resistance will result in greater overall resistance to FHB. This could be explored to improve the classification of new wheat cultivars from moderate resistance to a full resistance.

Two main mechanisms can explain the FHB resistance in wheat: physiological resistance (active) and disease escape (passive) (Mesterhazy, 1995). Physiological resistance decreases infection and reproduction of *F. graminearum* by active plant processes with gene products that contribute to plant defense; disease escape (or passive resistance) allows plants to avoid fungal infection and/or disease progression through morphological and developmental features (Mesterhazy, 1995; Prat et al., 2017). Numerous FHB resistance loci, identified as quantitative trait loci (QTL), are associated with developmental traits, such as heading date, flowering time, anther retention, plant height, and features of the wheat head that include spike length and spike density (Gervais et al., 2003; Srinivasachary et al., 2008, 2009; Buerstmayr et al., 2009; Skinnes et al., 2010; Lu et al., 2013; Szabo-Hever et al., 2014; Buerstmayr and Buerstmayr, 2016). These developmental traits contribute to FHB resistance through disease escape. A notable example is the well-documented negative association between plant height and FHB incidence and severity (Srinivasachary et al., 2008, 2009; Lu et al., 2013; Buerstmayr and Buerstmayr, 2016; He et al., 2016).

Progress in breeding FHB-resistant cultivars has been hindered by the lack of effective resistance sources and the quantitative nature of FHB resistance. FHB resistance is a polygenic trait controlled by multiple genes that can have either major or minor effects and is significantly affected by the interaction of genotype and environment (Bai and Shaner, 1994; Gilbert and Tekauz, 2000; McMullen et al., 2012; Buerstmayr et al., 2019). For hexaploid wheat, several highly resistant sources have been identified and exploited for breeding purposes, including the Chinese cultivars Sumai 3 and Wuhan 1, the Chinese landrace Wangshuibai, the Japanese landrace NyuBai, and the Brazilian cultivar Frontana (Bai and Shaner, 1994; Buerstmayr et al., 2019). However, use of alien germplasm is problematic, notably due to the introgression of undesired genes linked to resistance, known as linkage drag. In these cases, a main challenge is to achieve desirable FHB resistance while maintaining good yield, quality, and agronomic traits. The use of native QTL identified in adapted cultivars without associated linkage drag is therefore preferred by breeders to develop cultivars with better FHB resistance (McCartney et al., 2016; Steiner et al., 2017). Due to the smaller effects of known native QTL, multiple QTL need to be combined, or pyramided, to achieve a desirable level of resistance.

Genetic studies have identified as many as 556 QTL for FHB resistance on all 21 chromosomes in wheat (Venske et al., 2019). Meta-QTL analysis has consolidated these into 19–65 clusters (Liu et al., 2009; Löffler et al., 2009; Venske et al., 2019). Three of these, all originating from the very resistant cultivar Sumai 3, have been validated as major QTL contributing to FHB resistance (Buerstmayr et al., 2009, 2019), including *Fhb1* on chromosome arm 3BS (Anderson et al., 2001; Liu et al., 2006), Qfhs.ifa-5A on 5AS (*Fhb5*) (Buerstmayr et al., 2002; Somers et al., 2003; Xue et al., 2011; Steiner et al., 2019), and *Fhb2* on 6BS (Anderson et al., 2001; Cuthbert et al., 2007). All three of these major QTL have been exploited for wheat breeding, although most attention has focused on *Fhb1*, which is now present in several new FHB-resistant North American and European varieties (Hao et al., 2019). *Fhb1* accounts for 20–60% of phenotypic variation in breeding populations, primarily by conferring strong type II resistance (Miedaner and Korzun, 2012). It is the QTL most consistently reported in the literature and three groups have recently cloned *Fhb1* gene candidates, although they propose three different mechanisms of action for two different candidate genes (Rawat et al., 2016; Li et al., 2019; Su et al., 2019). The majority of FHB studies have focused on type I and II resistance, with relatively few studies of DON and FDK. Although toxin levels are ultimately the most important measure of disease to consumers, these studies have been limited largely due to the high costs of toxin analysis. The correlation between DON, FDK, and other types of FHB resistance can vary significantly and only a small number of QTL have been reported as exclusively associated with DON, including on 2AS (Semagn et al., 2007), 3A, 7B (Szabo-Hever et al., 2014), 3B, and 3D (He et al., 2019).

In the present study, we aimed to characterize and identify the genetic architecture of different FHB-resistant components including incidence, severity, FDK, and DON from a multi-parental population created by crossing FL62R1 with the two elite Canadian wheat cultivars Stettler and Muchmore. FL62R1 is a wheat line with good FHB resistance derived from the four-way cross QG22.24 / Alsen // SS Blomidon / Alsen that targeted to combine both type I and type II resistance from QG22.24 and Alsen, respectively (Comeau et al., 2008). The line FL62R1 was selected by a “systemic genetic breeding approach” implemented in Eastern Canada (Comeau et al., 2008). It has comparable FHB resistance to the best check, Sumai 3, but possesses good yield potential and agronomic traits (Comeau et al., 2008). Characterization of the resistant QTL for different resistance components from FL62R1 will facilitate achieving desirable FHB resistance by combining these resistance components. In addition, recent findings indicate that FHB resistance QTL are often associated with morphology and development-related genes, such as plant height and flower time (Mesterhazy, 1995; Prat et al., 2017; reviewed by Steiner et al., 2017). In the present study, we further investigated this correlation in different genetic backgrounds and multiple environments. Information from this analysis may provide an approach to incorporate traits related to disease escape into the management of FHB resistance by breeding practices.

## Materials and Methods

### Materials

Two spring wheat (*Triticum aestivum* L.) double haploid (DH) populations, derived from the cross of FL62R1 (as common parental line) with Stettler (Stettler population) and Muchmore (Muchmore population), were developed using microspore culture (Eudes and Amundsen, 2005). FL62R1 is an Eastern Canadian spring wheat line derived from the systemic breeding approach, where it was selected from the four-way cross QG22.24 / Alsen // SS Blomidon / Alsen that was subjected to complex stresses over for a few generations (Comeau et al., 2008). The parent Blomidon with pedigree “Weih23.1/Kokart” was bred in Germany, and the QG22.24 parent with the pedigree “5thLACOS-167 / Ae. kotschyi, 400008 // AC Pollet /3/ 5thLACOS-167,” from eastern Canada, showed type I FHB resistance (personal communication with wheat breeders, Drs. A. Comeau and S. B. Rosa). Stettler and Muchmore are two semi-dwarf, high-yielding Canada Western Red Spring (CWRS) wheat cultivars (DePauw et al., 2009, 2011a). The Stettler population contained 182 lines and the Muchmore population consisted of 202 DH lines.

### Disease Inoculation and Phenotyping

The two DH populations were evaluated for FHB resistance in disease nurseries at Ottawa, Ontario, and Carman, Manitoba in 2015 and 2016 with three biological replications and a random complete block design (RCBD) in single meter rows. At the Ottawa nursery, grain spawn inoculation was used, as described by Xue et al. (2006) and McCartney et al. (2016). Briefly, a mixture of three local *F. graminearum* isolates (Xue et al., 2006; McCartney et al., 2016) were used to infect corn and barley kernels, which were spread on the soil surface at a rate of 80 g m^2^ 6 weeks after planting. To promote FHB infection, plots were irrigated for 30 min every morning and 30 min each afternoon to create favorable moisture for infection (Xue et al., 2006; McCartney et al., 2016).

A spray approach was used to inoculate plants at the Carman disease nursery. When ∼50% of plants reached anthesis, a mixture of four local *F. graminearum* isolates were applied at a rate of 50 ml per row and sprayed onto plants at a concentration of 50,000 macroconidia L^–1^. Plants were sprayed a second time 2–3 days after the first application. Thereafter, a daily mist irrigation for 10 min every hour for a 12-h period (6:00 pm–6:00 am) was utilized to promote FHB infection (McCartney et al., 2016).

At 18–21 days post-inoculation, FHB incidence (INC) was scored as an estimate of the percentage of infected heads within the plot, and severity (SEV) was measured as an estimate of infected spikelets in an infected head. Visual rating index (VRI) was estimated from INC and SEV using the formula: VRI = (INC × SEV)/100. Plants were harvested with minimum air force to prevent the loss of low weight infected kernels and a visual assessment of kernel damage in the collected seed was used to estimate kernel damage, expressed as percentage of FDK. The concentration of DON in grain was evaluated by commercial enzyme linked immunosorbent assay (ELISA) kits, Neogen Veratox^®^ (Lansing, MI, United States) for DON 5/5 ELISA kits (Ye, 2015). In addition, measurements of days to anthesis (DA) was recorded when 50% of the main tillers in the row had begun anthesis and plant height (HT) was measured from the soil surface to the top of main tiller spikes.

### Genotyping

Genomic DNA was extracted from the two DH populations using the BioSprint 96 Extraction Platform and DNA Plant Kit (Qiagen, Hilden, Germany). DNA was resuspended in purified water and quantified with a Quant-iT^TM^ PicoGreen^®^ dsDNA Assay Kit (Thermo Fisher Scientific Inc., Bartlesville, OK, United States). All DNA samples were diluted to 50 ng/μl for SNP array genotyping. The two DH populations were genotyped with the Illumina iSelect 90K SNP array (Wang et al., 2014) and raw data were processed and cleaned as described by Wang et al. (2014) using the diploid version of GenomeStudio (Illumina, San Diego, CA, United States). The SNP marker to differentiate *Rht-B1* semi dwarf allele was run following standard KASP guidelines (Rasheed et al., 2016). Additionally, 423 SSR markers were tested, including some previously reported as associated with FHB QTLs and randomly selected markers that spanned every chromosome. Markers that showed clear polymorphism and segregation were selected and added to the SNP map. High-quality SNP and SSR markers were further refined with the R/QTL package (Broman et al., 2003) using the following criteria: heterozygous SNPs were converted to missing data; SNPs with missing genotypic data higher than 15% and/or individuals with more than 20% missing genotypic data were removed; SNPs and SSRs with the minor allele frequency less than 10% were also discarded from downstream analysis.

### QTL Mapping

Genetic maps were developed with Mapdisto (Lorieux, 2012) using the markers described above, a cutoff recombination value of 0.3, and a threshold logarithm of odds (LOD) of 6.5. Physical positions of mapped markers were retrieved by blast searching SNP sequences against IWGSC RefSeq v1.0 (International Wheat Genome Sequencing Consortium, 2018; CS Ref 1.0) with best hits. QTL analysis was performed by composite interval mapping (CIM) as implemented in WinQTL Cartographer (Wang et al., 2007) with a walking speed of 1 cM and a window size of 10 cM. The significance of a QTL was claimed at the threshold of 5% significance level by the 3000 permutation test in WinQTL Cartographer (Churchill and Doerge, 1994; Basten et al., 2001; Wang et al., 2007). Due to the known minor effects of most FHB resistance-related QTL, QTL with an LOD value below the 5% significance level but higher than the empirical cutoff threshold value of LOD = 2.5 were also included in the analysis. QTL were physically projected on CS Ref 1.0 (International Wheat Genome Sequencing Consortium, 2018) using the physical position of peak markers and were visualized by the R package RIdeogram (Hao et al., 2020).

### Statistical Analysis

Statistical analysis of the available phenotypic data was performed in R 3.30 (R Core Team, 2014) with the lme4 package (Bates et al., 2015). A linear mixed model analysis (Bates et al., 2015) was fitted for each phenotypic trait. For within-year data analysis, the linear model used was y_ib_ = μ + G_i_ + R_b_ + e_ib_, where y_ib_ is the trait, G_i_ is the effect of the *i*-th genotype in the *b*-th block, μ is the mean value, R_b_ is the effect of the *b*-th block, and e_ib_ is the residual. For across years, the model used was y_ijb_ = μ + G_i_ + E_j_ + (GXE)_ij_ + r_(j)b_ + e_ijb_, where, y_ijb_ is the phenotypic value of *i*-th genotype in the *k*-th year and *b*-th block, μ is the mean value, G_i_ is the effect of the *i*-th genotype, E_j_ is the effect of the *j*-th year, (GXE)_ij_ is the effect genotype-by-year interaction of the *i*-th genotype in *j*-th year, r_(j)__b_ is the effect of *b*-th block in the *j*-th year, and e_ijb_ is the residual. The variance component of each trait was determined by the restricted maximum likelihood (REML) method within lme4 (Bates et al., 2015) by setting all effects as random. Variance components were used to estimate the repeatability within year with the equation $H^{2}=\frac{\delta_{G}^{2}}{\left(\delta_{G}^{2}+\frac{\delta_{e}^{2}}{b}\right)}$ and broad-sense heritability across year was estimated using the equation $H^{2}=\frac{\delta_{G}^{2}}{\left(\delta_{G}^{2}+\frac{\delta_{GXE}^{2}}{j}+\frac{\delta_{e}^{2}}{jb}\right)\;}$, where $\delta_{G}^{2}$, $\delta_{GXE}^{2}$, and $\delta_{e}^{2}$ stand for variance of genotype, genotype-by-year interactions, and error; *j* represents number of years, and *b* indicates number of blocks.

## Results

### Phenotypic Analysis

**FIGURE 1:**
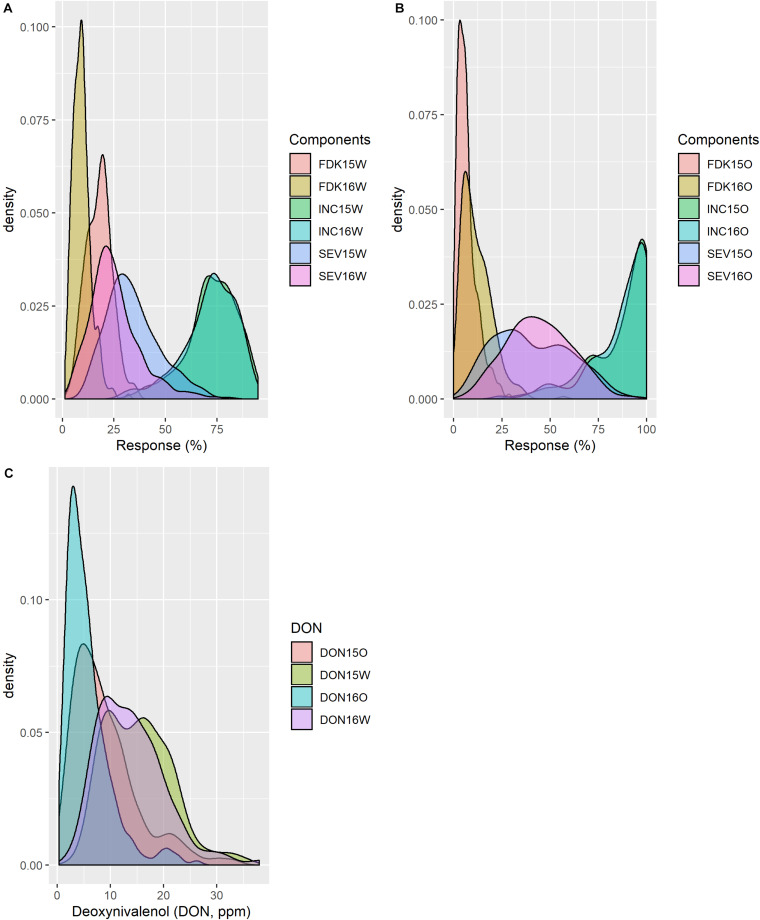
Distribution of phenotypic traits from field trials in different years and locations. Fusarium damaged kernels (FDKs), incidence (INC), and severity (SEV) at **(A)** Carman, Manitoba (W) and **(B)** Ottawa, Ontario (O) trials; and **(C)** deoxynivalenol (DON) from all environments.

Considering all lines from the two populations analyzed, INC ranged from 0 to 100% at both Carman and Ottawa with a mean value of approximately 70–80% (Table 1 and Figure 1). The distributed SEV observed at Carman ranged from 5 to 95%, skewed toward a lower mean value of around 30%, while at Ottawa, SEV had a near-normal distribution, with a mean around 45%. FDK was observed from 1 to 47% with a mean of approximately 15% at both sites (Table 1 and Figure 1). DON levels ranged from 0.1 to 47.0 ppm with distribution toward a lower mean value of about 10 ppm at both sites (Table 1 and Figure 1). DA and HT have high heritability and show normal distribution (Table 1 and Supplementary Figure 1). All traits tested showed moderate to high heritability, with *H*^2^ values from 0.55 to 0.88 (Table 1).

**TABLE 1:** Phenotypic variation and heritability (*H*^2^) of Fusarium head blight (FHB) and plant traits from field experiments of the Muchmore and Stettler populations in different environments

Location	Trait	Pop	Year	Range	Mean	*H*^2^	Location	Trait	Pop	Year	Range	Mean	*H*^2^
Ottawa	DA (days)	MM	15_16	39–61	50.05	0.791	Carman	DA	MM	15_16	38–55	45.11	0.876
			15	45–61	50.9	0.845				15	41–48	45.0	0.862
			16	39–61	49.2	0.747				16	38–55	45.2	0.902
		ST	15_16	39–72	51.31	0.803			ST	15_16	38–57	44.65	0.740
			15	45–66	52.1	0.899				15	38–50	44.0	0.774
			16	39–72	50.5	0.846				16	41–57	45.3	0.895
	DON (ppm)	MM	15_16	0.2–45.7	7.35	0.641		DON	MM	15_16	3.4–38	13.70	NA
			15	0.3–45.7	8.3	0.764				15	5.5–29	14.48	NA
			16	0.2–41.5	6.2	0.672				16	3.4–38	12.92	NA
		ST	15_16	0.1–43.5	7.39	0.596			ST	15_16	2.6–42	16.41	NA
			15	0.25–43.5	9.5	0.845				15	2.7–42	16.77	NA
			16	0.1–33.3	5.3	0.583				16	2.6–39	16.043	NA
	FDK (%)	MM	15_16	0–49	10.18	0.589		FDK	MM	15_16	1.3–39.3	12.70	NA
			15	0–50	8.1	0.645				15	3.3–39.3	16.31	NA
			16	0–47	12.3	0.728				16	1.34–3	9.098	NA
		ST	15_16	0–47	8.54	0.590			ST	15_16	2.18–35.9	15.22	NA
			15	0–34	5.5	0.747				15	3.7–35.9	20.33	NA
			16	0–47	11.6	0.648				16	2.18–31.9	10.105	NA
	HT (cm)	MM	15_16	47–110	81.18	0.800		HT	MM	15_16	NA	NA	NA
			15	60–110	88.8	0.844				15	NA	NA	NA
			16	47–108	73.6	0.739				16	50–116.7	92.7	0.858
		ST	15_16	56.5–113.5	81.02	0.879			ST	15_16	NA	NA	NA
			15	66–120	93.9	0.886				15	NA	NA	NA
			16	47–107	78.1	0.814				16	60–121.7	95.4	0.889
	INC (%)	MM	15_16	10–100	87.47	0.555		INC	MM	15_16	20–100	71.65	0.648
			15	20–100	88.8	0.662				15	20–100	72.0	0.697
			16	10–100	85.5	0.480				16	20–95	71.3	0.885
		ST	15_16	10–100	85.91	0.672			ST	15_16	10.1–100	74.83	0.760
			15	15–100	85.2	0.719				15	10–95	75.3	0.600
			16	10–100	86.6	0.738				16	30–100	74.4	0.874
	SEV (%)	MM	15_16	5–100	44.76	0.620		SEV	MM	15_16	5–85	27.37	0.700
			15	5–100	44.2	0.769				15	5–85	29.5	0.708
			16	5–95	45.4	0.698				16	5–80	25.2	0.844
		ST	15_16	5–100	40.19	0.821			ST	15_16	5–95	34.17	0.849
			15	5–100	37.3	0.851				15	5–95	40.9	0.695
			16	5–100	43.1	0.875				16	5–70	27.4	0.875

*INC, incidence; SEV, severity; FDK, Fusarium damaged kernels; DON, deoxynivalenol; HT, plant height; DA, day to anthesis; O, Ottawa, Ontario; C, Carman, Manitoba. 15, 2015; 16, 2016; ST, Stettler population; MM, Muchmore population.*

**FIGURE 2:**
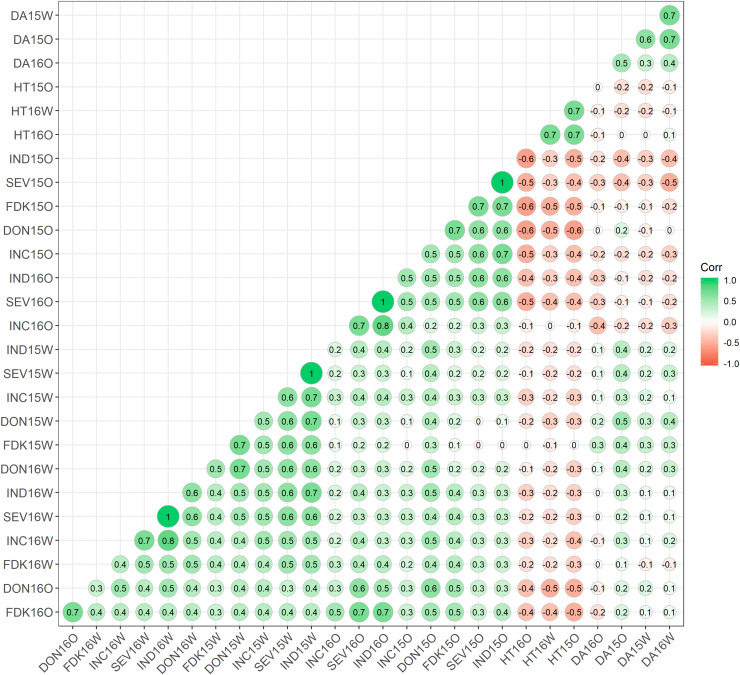
Phenotypic correlation between Fusarium head blight-resistance components and associated traits at two field locations for two populations. The areas of the circles show the absolute value of the corresponding correlation coefficients (*r*). *INC*, incidence; *SEV*, severity; *FDK*, Fusarium damaged kernels; *DON*, deoxynivalenol; *HT*, plant height; *DA*, day to anthesis; *O*, Ottawa, Ontario; *W*, Carman, Manitoba. *15*, 2015; *16*, 2016; *ST*, Stettler population; *MM*, Muchmore population.

Positive correlation was observed among all FHB resistance components, while DA and HT showed mostly negative correlation with FHB traits (Figure 2). When comparing the same location and year, INC, FDK, SEV, and DON correlated well with each other (*r* = 0.4–0.7). Correlations were still high among these four resistant components when comparing across 2 years at the same location, with *r* ranging from 0.3 to 0.7. However, the correlation between FHB resistance components was lower across sites, with moderate *r* ranging from 0.3 to 0.5 across all environments. When comparing the individual FHB measures, the visual disease resistance components (INC, SEV, and VRI) were generally better correlated with each other than with FDK and DON, and FDK and DON were very highly correlated with each other (Figure 2). A high negative correlation was observed between HT and all the resistance components at Ottawa in both years, ranging from −0.3 to −0.6. However, a substantially lower correlation was found for these traits at Carman (from 0 to 0.3). DA displayed the lowest correlation against the resistance components, with *r* values from −0.2 to 0.4. The HT and DA traits showed the highest correlation across all the tested environments with *r* values from 0.5 to 0.7, respectively.

The correlation between greenhouse type II resistance in the Stettler population (Zhang et al., 2018) and field resistance was also examined (Supplementary Figure 2). The type II FHB resistance measured in the greenhouse showed significant but lower correlations with SEV in all tested environments (*r* = 0.23–0.37; Supplementary Figure 2). At Carman, except DON in 2015, INC, FDK, and DON also showed significant but lower correlations with greenhouse resistance (Supplementary Figures 2A,C). However, there was no correlation between the greenhouse results and Ottawa trials except for a small but significant correlation with 2015 FDK and DON (Supplementary Figures 2B,D).

### QTL Analysis

**FIGURE 3:**
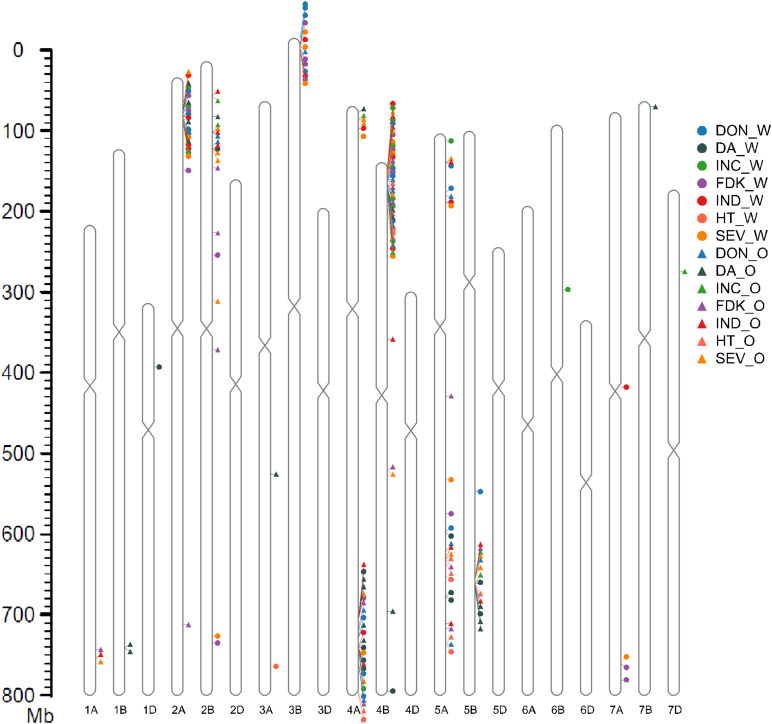
QTLs of Fusarium head blight resistance, flower time, and plant height anchored on the Chinese Spring reference 1.0 (CS Ref 1.0). The marker located at the peak of the QTL identified by blast on CS Ref 1.0 was used to localize QTL. *INC*, incidence; *SEV*, severity; *FDK*, Fusarium damaged kernels; *DON*, deoxynivalenol; *HT*, plant height; *DA*, day to anthesis; *O*, Ottawa, Ontario; *W*, Carman, Manitoba.

Quantitative trait loci analysis identified a large number of loci that contributed the different resistance components (Table 2 and Supplementary Table 1). Using the IWGS Chinese Spring (CS) reference 1.0 (CS Ref 1.0; International Wheat Genome Sequencing Consortium, 2018), the physical position of the QTL was anchored to approximate genomic regions. This permitted us to reduce the large number of QTL down to nine genomic loci affecting all of the FHB resistance components, located on chromosome arms 2AS, 2BS, 3BS, 4AS, 4AL, 4BS, 5AS, 5AL, and 5B (Table 2 and Figure 3). The majority of the FHB resistance QTL were contributed by the common parental line FL62R1, and generally, this line was also associated with increased HT and delayed DA. Detailed information on QTL detected is provided below, prioritized by their contribution to FHB resistance.

**TABLE 2:** Nine major QTL identified for FHB-resistant components across four environments in the Muchmore and Stettler populations.

QTLChr^a^	2AS			2BS			3BS			4AS			4AL*			4BS			5AS			5AL			5BL		
Pos (cM)^b^	38.7–73.9			1.3–44.6			5.2–14.4			20–65			23–37; 163–216			5–52			97.7–213			75–92			60–113		
PhysPos (Mb)^c^	37–55			42–364			8.8–16			6.9–39			641–742			8.4–223			416–657			33–86			448–583		
	LOD^d^	Ad^e^	PV^f^	LOD	Ad	PV	LOD	Ad	PV	LOD	Ad	PV	LOD	Ad	PV	LOD	Ad	PV	LOD	Ad	PV	LOD	Ad	PV	LOD	Ad	PV
**Deoxynivalenol**																											
MO15				7.0	1.8	10							2.8	1.2	4.9	2.9	2.9	27	6.7	1.7	11						
MO16																9.4	2.4	20				7.4	2	16			
MW15	9.2	1.9	13				6.5	1.6	8.7				7.2	−1.7	9.7	8.7	2	13	5	1.4	6.9						
MW16	5.8	1.5	9				4.2	1.3	6.9				3.4	−1.2	5.9	4.4	1.2	6.3	3.4	1.1	5.2						
SO15							4.1	2	6.7				3.5	1.7	4.5	13	3.9	24				6.5	2.5	9.8			
SO16																12	1.9	28							2.7	−0.9	5.2
SW15	3.8	1.7	5.9				8.8	2.7	15				5.1	−2	7.9	6.7	2.3	11									
SW16	4.6	2	7.2				11	3.2	19							7.7	2.6	12				3.5	1.7	5.3	2.6	1.4	3.7
**Fusarium damaged kernels**																											
MO15				3.3	1.4	6.7							4.1	1.6	8.5	13	3.3	35				4.4	4	8.9			
MO16				14	3.4	14													6.9	3.2	13						
MW15	6.9	2	11				11	2.7	18				4.1	−1.6	6.1												
MW16	2.7	0.8	3.9	6.9	2.1	11	9.3	1.6	15							3.5	1	5.4				4.4	1.1	6.8			
SO15													4.9	4.9	8.5	5.8	2	10				4	1.3	7.1	4.8	−1.5	8.4
SO16																12	3.8	27									
SW15							6.8	2.3	13							2.9	1.5	5.4									
SW16							8.8	1.9	15							5.5	1.5	9.4									
**Incidence**																											
MO15				2.7	3.5	6.7										5.5	5.3	14									
MO16				3.5	4.8	9.4																					
MW15	3.2	2.9	5.4										3.3	−2.9	5.4	8.4	5.1	16									
MW16																4.9	4	8.5									
SO15										5.4	−5.1	9.2				7.9	6.5	15							7.6	−6.3	13
SO16																									7.3	−7.1	17
SW15	3.7	2.9	5.1													19	5.9	19									
SW16	2.8	2.3	3.5													13	6.9	8.7	4.2	2.8	5.4						
**Index**																											
MO15				5.6	6.9	10							7	7.7	14	3.6	5.8	7.8				3.3	5.1	6.4			
MO16				5	5.9	11													2.5	4.5	6.9						
MW15	3.2	2.5	5.2										6.6	−3.7	11	3.2	2.5	4.9									
MW16	2.5	2	4.1				3.4	2.4	5.6										4.3	4.3	7.1						
SO15													8.7	8.2	13	3.4	6.6	5.1				4.3	4.3	3.5	12	−10	20
SO16																									10	−10	23
SW15	4	3.6	6.3				2.9	3.1	4.6							4.2	4.5	7.1									
SW16	4.3	3.8	7.6							2.5	−2.8	4.1				3.6	4	5.8									
**Severity**																											
MO15				5.6	6.4	11							6.6	7	14	5.8	6.5	12				3.5	4.9	6.9			
MO16	3.5	4.3	7.7	8.3	3.7	7													4.2	5.1	11						
MW15							6.4	3.7	10				8.2	−4.2	14	4.6	3.1	7.3				2.8	2.4	4.5			
MW16							3.9	2.6	6.7										3.8	2.6	6.5						
SO15										4.3	−5.5	6.5	8.8	7.9	14	3.8	5.9	5.6							12	−9.8	20
SO16										2.6	−4.2	5.2				8.1	7.8	17							10	−9	23
SW15	2.6	2.9	4.2				4.2	4.2	6.7							8.4	5.5	15									
SW16	4	3.9	7.1							3.5	−3.5	5.8				8.3	5.7	15									
**Days to anthesis**																											
MO15	6.6	0.7	9	2.7	−0.4	3.3							20	−1.3	35												
MO16	2.5	0.7	6.2										5.4	−1.1	14												
MW15													36	−1.3	50							6.3	0.5	6.5			
MW16				4.9	−0.6	4.4							38	−1.9	49							3.7	0.5	3.2			
SO15	3.4	0.6	4.9							4.1	0.7	6.4	6.7	−0.9	11	6	0.9	9.8							6.6	1	11
SO16																									7.6	0.2	17
SW15													19	−0.8	22							3.4	−0.3	4.2	7	0.4	9.2
SW16													32	−0.9	11										12	1.3	20
**Plant height**																											
MO15																30	−7.6	58				2.7	−1.6	2.9			
MO16													5.6	−2.6	9.1	16	−5.4	38				5.3	−2.5	8.6			
MW16													2.9	−1.8	3.1	32	−7.1	50				5.2	−2.4	5.6			
SO16																27	−7.2	50				2.8	−2	4.4	6.7	2.8	8.8
SW16																37	−7.5	57				6.4	−2.6	7			

^a^QTL defined as chromosome (chr.) location.^b^Pos, position on linkage group (cM).^c^Physical position of peak markers on based on IWGSC 2018; doi.org/10.1126/science.aar7191.^d^LOD, peak LOD score.^e^PV, phenotypic variation explained (r2; %).^f^Ad, additive effect of QTL; negative values indicate that the “FL62R1” allele increased the respective quantitative trait, and positive values indicate that the Muchmore or Stettler parent increased the trait scores. *4A linkage group in Stettler population was broken into two linkage groups: 4AS and 4AL, and the interval 23–37 is from the 4AL linkage group. M, Muchmore population; S, Stettler population; W, Carman, Manitoba; O, Ottawa, Ontario; 15, 2015; 16, 2016.

4BS: The 4BS genomic locus, from 7.04 to 42.02 Mb on the CS Ref 1.0 assembly, is the region with the highest number of QTL identified in this study. It was detected across almost all environments for the FHB resistance traits and plant height (Table 2 and Figure 3). The phenotypic effect of the 4BS region on the different resistance components was greater than observed for other QTL identified, with the percentage of phenotypic variance explained by the QTL (PV) ranging from 5.4 to 28% (Table 2). The 4BS locus had a more stable effect in the Stettler population compared to the Muchmore population. We also observed that 4BS contributed more to FDK and DON resistance at Ottawa than at Carman, with approximately twofold higher PV in both populations in 2 years of field trials. At Ottawa in 2015, 4BS QTL were found to have the largest effect on all FHB resistance components, with PV of 35.4 and 27.4% for FDK and DON, respectively. Mapping of the functional *Rht-B1* KASP marker (Rasheed et al., 2016) and anchoring to the CS Ref 1.0 assembly located the 4BS QTL to the chromosomal region carrying *Rht-B1*.

3BS: QTL identified on 3BS were localized to an interval between 6.68 and 11.58 Mb on the CS Ref 1.0 assembly. QTL for SEV, FDK, and DON were stably detected in both populations at Carman in 2015 with relatively large effects, with PV of 7–19%. During the 2016 trial, QTL also were found for these components except for SEV in the Stettler population. No QTL was found for INC in this genomic locus. QTL for VRI were identified in the Stettler population in 2015 and the Muchmore population in 2016. At Ottawa, the only QTL in this region was identified for DON in the 2015 Stettler population. No DA or HT QTL were found in the 3BS region.

4AL and 4AS: The QTL from 4AL (physical position from 641.4 to 692.4 Mb on the CS assembly) were mainly detected in the 2015 trials. For the Muchmore population, stable QTL were found at both Carman and Ottawa for SEV, FDK, DON, and VRI, while QTL for INC were only detected at Carman in this region. For the Stettler population, QTL were also found for SEV, FDK, DON, and VRI on 4AL at Ottawa, while this region only had a QTL for DON at Carman. In 2016 the 4AL region had a smaller impact on FHB resistance, with only a minor effect for DON QTL found in the Muchmore population at Carman. One of the largest and most significant QTL in the entire dataset was found for DA in the 4AL region. It was detected in each population, year, and location, except for Stettler at Ottawa in 2016, and had a large impact with PV ranging from 10.9 to 49.8%. The common parental line, FL62R1, contributed to longer DA and taller plants, though at Ottawa, the locus contributed to FHB resistance but increased FHB susceptibility at Carman.

In the region on 4AS (∼12.2–38.7 Mb), QTL were only detected in the Stettler population. SEV was the most stable QTL detected at Ottawa and Carman. The alleles contributed by Stettler conferred better disease resistance at the 4AS QTL.

5AL and 5AS: The 5AL genomic locus covered the region from 528.6 to 584.7 Mb on the CS Ref 1.0 assembly. QTL from this region were observed for all of the traits, though each was not consistent across environments. The largest effect 5AL QTL were for DON at Ottawa; the Muchmore population in 2016 with a PV of 16%, followed by a 9.8% PV from the Stettler population in 2015. Minor QTL for DA and HT were found on the 5AL region in both populations.

FHB resistance QTL were found on 5AS in the Muchmore population only. In 2015, 5AS QTL were found in Ottawa and Carman (contributing up to 11% PV). In 2016, a 5AS QTL for DON was detected in Carman, in addition to an FDK QTL at Ottawa. VRI and SEV 5AS QTL were found at both sites in 2016.

5BL: QTL from this region were located from 551.0 to 582.5 Mb in the CS 1.0 reference genome. The 5BL QTL were only identified in the Stettler population, where they were responsible for a large phenotypic effect on FHB resistance at Ottawa in 2015 and 2016, with PV as high as 13.3% for INC and 22.7% for SEV. In all cases, alleles from Stettler contributed to disease resistance at the 5BL locus. A major QTL with large effect for DA was detected in the 5BL interval in the Stettler population, with Stettler responsible for delayed anthesis.

2AS and 2BS: The 2AS QTL were localized from 36.6 to 58.4 Mb on the CS Ref 1.0 assembly. This region conferred resistance for almost all of the resistance components in both the Stettler and Muchmore populations in both years of testing at the Carman site. No QTL was detected on 2AS at Ottawa.

The 2BS QTL region controlled the majority of FHB resistance traits in the Muchmore population at Ottawa in 2015 and 2016.

## Discussion

FHB resistance in wheat is a complex trait and controlled by several resistance components including INC, SEV, DON accumulation, FDK, and spreading (greenhouse). A better understanding of the genetic architecture underlying these different components may enable the development of wheat cultivars with better resistance to FHB. In the present study, a systematic analysis of different components was undertaken with two large bi-parental mapping populations, revealing how different FHB resistance components, plant height and flowering time, interact.

### Correlation Between FHB Resistance Component Traits

There was a moderate to high heritability displayed across the FHB resistance components tested, which is in agreement with previous findings, indicating that genetic variation plays a main role in the phenotypic variation of these FHB traits (Buerstmayr et al., 2000; Lu et al., 2013; He et al., 2019). In contrast, the highly complex genotype-by-environment interactions of FHB resistance are emphasized with the low to moderate correlation observed between the different FHB resistance components measured in this study (INC, SEV, FDK, and DON) when comparing across years and sites. Visual disease symptoms were highly correlated, as were FDK and DON; however, INC and SEV were found to have lower correlation with DON and FDK (Figure 2A). Our results agree with previous findings (Paul et al., 2005; Lu et al., 2013; He et al., 2019) that visual FHB disease symptoms are poorly correlated with DON content, indicating that visual FHB disease resistance contributes only in part to DON accumulation. Given the increasing priority for breeding new varieties with greater resistance to DON, Canadian cultivar registration is evaluated with an index (ISD = 0.2 × INC + 0.2 × SEV + 0.6 × DON) that gives greater weight to DON accumulation than visual measures of disease resistance combined^2^. There is a clear need to understand the genetic architecture of the different resistance components beyond INC and SEV.

FHB type II resistance (resistance to fungal spread in the spike) is determined by point inoculation in the greenhouse, while SEV is routinely used as an estimate for field FHB spread/type II resistance (Bai and Shaner, 2004). There was a significant but low correlation between greenhouse type II resistance using our previously published data (Zhang et al., 2018) and SEV in the present study (Supplementary Figure 2). Lu et al. (2013) observed a lack of correlation between greenhouse and field FHB resistance in their germplasm, reasoning that this discrepancy is due to a lack of *Fhb1*, a gene commonly reported as conferring major type II resistance. FL62R1 is moderately susceptible to point inoculation despite carrying *Fhb1* (Zhang et al., 2018), supporting the interpretation from Lu et al. (2013). However, the major 2BL QTL controlling greenhouse type II resistance identified by Zhang et al. (2018) was not detected by field evaluation, indicating that greenhouse type II resistance may occur through a somewhat different mechanism than field resistance in the populations analyzed in this study.

A higher negative correlation was observed between plant height and FHB resistance components at Ottawa than at Carman. This is likely the result of the different FHB inoculation approaches utilized at each location: grain spawn at Ottawa and spray treatment at Carmen. The spawn approach relies on wind or water splashes to spread FHB spores from the ground surface to spikes and establish infection (McMullen et al., 2012). The spray approach directly applies FHB spores onto wheat spikes, thus minimizing the effect of height on FHB infection caused by the different disease pressure at different distances between the spike and soil surface. This may explain the low negative correlation between plant height and FHB resistance components at Carman. However, taller plants are also prone to have a less favorable environment (less moisture) for disease infection and development (Yan et al., 2011), and neither spawn nor spray approach can differentiate the effect of height on FHB resistance related to the different spike microclimates. The strong negative correlation between FHB resistance and plant height and flowering time is well established (Buerstmayr et al., 2009; Buerstmayr and Buerstmayr, 2016) and discussed in greater detail below.

### Genetic Architecture of FHB Resistance Components and Their Association With Flowering Time and Plant Height

A large number of loci that control different FHB resistance were identified, with genomic loci on 2AS, 2BS, 3BS, 4AS, 4AL, 4BS, 5AS, 5AL, and 5BL enriched with QTL for one or more traits. The genomic locus 4BS from FL62R1 conferred the most stable FHB resistance, with INC, SEV, FDK, and DON QTL identified at all tested environments, in both the Stettler and Muchmore populations. A major QTL for plant height was also identified in this region of 4BS that can be attributed to the presence of the semi-dwarf allele of *Rht-B1*. Numerous studies have previously demonstrated that semi-dwarf *Rht1* alleles are associated with FHB susceptibility (Srinivasachary et al., 2008, 2009; Lu et al., 2013; Buerstmayr and Buerstmayr, 2016; Prat et al., 2017), with the wild-type alleles *Rht-B1a* and *Rht-D1a* contributing to FHB resistance. *Rht-B1* may contribute to resistance directly through the control of the plant height and flower microclimate through pleiotropy, hormone signaling, and modulation of flowering, anther retention and spike traits, or even through linkage drag (McCartney et al., 2007; Yan et al., 2011; Buerstmayr and Buerstmayr, 2016; Davière and Achard, 2016; He et al., 2016; Herter et al., 2018; Xu et al., 2020). The present study found that when the effect of plant height on FHB resistance caused by different disease pressure was controlled through the use of spray inoculation at Carman, the 4BS locus still had a large effect on FHB resistance, though the effect was reduced compared to the site using the spawn approach. The effect caused by different microclimates of spikes at different heights was not investigated in this study, so height differences per se could still be responsible for the FHB resistance observed, though other mechanisms are likely also involved. Future application of CRISPR technology to edit wild-type *Rht-B1* alleles will likely prove very useful to resolve the association between FHB resistance and *Rht-B1* dwarf alleles.

The largest effect and most extensively characterized FHB resistance QTL is *Fhb1* located on chromosome arm 3BS (Anderson et al., 2001; Buerstmayr et al., 2019; Hao et al., 2019), which was also identified in this study. We found that the *Fhb1* QTL from the common parental line FL62R1 affected SEV, VRI, FDK, and DON, with a large phenotypic effect on FDK and DON at Carman in both populations. *Fhb1* was likely contributed from Alsen, one of the parental lines of FL62R1 (Comeau et al., 2008). Alsen was the first North American high-quality hard red spring cultivar with improved FHB resistance (Frohberg et al., 2006). *Fhb1* has been proposed as the key factor for DON detoxification (Lemmens et al., 2005), and our results support this claim. In spite of *Fhb1* being important to type II FHB resistance, our previous study (Zhang et al., 2018) found that *Fhb1* from FL62R1 had no effect in the Stettler population during greenhouse point inoculation tests. This may be attributed to *Fhb1* having distinct effects when in different genetic backgrounds (McCartney et al., 2007; Bokore et al., 2017; Li et al., 2019; Brar et al., 2019). Most research has assessed the effect of *Fhb1* on non-adapted or adapted germplasm, with few studies conducted on elite cultivars. Indeed, testing of additional Canadian elite cultivars, such as Carberry (DePauw et al., 2011b) and Penhold (Cuthbert et al., 2017), corroborates the moderate susceptibility demonstrated in greenhouse point inoculation tests (L. P. Wang, unpublished). However, it is also possible that the heavy inoculum used in our greenhouse inoculation tests overwhelmed the plants and prevented the detection of any *Fhb1* effect.

In addition to genetic background, the method of inoculation, the aggressiveness of the *F. graminearum* race tested, the presence of other resistance genes, and environmental conditions are factors that could affect the level of resistance conferred by *Fhb1*. As pointed out by Hao et al. (2019), the different stages of development for inoculation and the aggressiveness of races used in *Fhb1* cloning experiments (Rawat et al., 2016; Li et al., 2019; Su et al., 2019) may be partially responsible for the different findings of these studies. The present study applied the spawn method of inoculation at Ottawa and spray inoculation at Carman. The aggressiveness of the races tested at each location differed, and the *Fhb1* QTL was only detected at Carman. It is possible that the stronger effect of the 4BS QTL at Ottawa, associated to plant height and thus more influenced by the inoculation method used, masked the detection of *Fhb1*. Liu et al. (2019) reported that *Fhb1* had a very minor effect in a hard red spring wheat breeding population in disease nurseries that used the spawn approach. Although these authors did not compare the effectiveness of *Fhb1* using spray inoculation, their findings are consistent with results we observed at Ottawa with spawn inoculation. Finally, Bokore et al. (2017) reported different effectiveness of *Fhb1* in Sumai 3-derived North American spring wheat breeding lines when tested at two locations.

The 5AS QTL was only found in the Muchmore population and showed variable effects on different resistance components. As observed for 3BS (*Fhb1*), no height- or flowering time-related QTL were detected in this region. The 5AS QTL harbors the SSR marker GWM304 that is linked to *Fhb5*, indicating that it may be Qfhs.ifa-5A (*Fhb5*). *Fhb5* has been reported to contribute mainly to INC and SEV when tested using the spray approach (Buerstmayr et al., 2002; Somers et al., 2003; Steiner et al., 2019). In the present study, the 5AS QTL showed minor effects for SEV, FDK, and DON in a portion of years and locations tested. The 5AS resistance allele is likely derived from Alsen through FL62R1. The Canadian CWRS cultivar Carberry has notably higher FHB resistance for its class, and also carries the *Fhb5* allele derived from Alsen (Bokore et al., 2017). These findings indicate that in addition to *Fhb1*, *Fhb5* was also deployed into some North American wheat cultivars. Because of its relatively minor effect, retaining this allele through visual section will be challenging, and marker-assisted selection could be a useful approach to introduce it to new cultivars and breeding programs.

The remaining genomic loci controlling FHB resistance (2AS, 2BS, 4AS, 4AL, 5AL, and 5BL) identified in this study were all found to colocalize with flowering time or flowering time and plant height. The large number of FHB resistance loci associated with flowering time indicates that a complex flowering regulatory network was involved in controlling disease resistance in the populations tested. The establishment of FHB infection needs a favorable environment for disease establishment and the appropriate susceptible stage of plant development, namely, the early flowering stage at the time of fungal introduction (Mesterhazy, 1995). Thus, if wheat plants do not flower during the period that offers favorable conditions for FHB infection created by the local environment, they will show reduced disease symptoms. Flowering time is largely thought of as controlling FHB infection through disease escape. However, it has recently become clear that the flowering period is also a key factor for wheat spikelet development, with many pleiotropic genes/QTL influencing the timing of flowering and development of spikes (Lewis et al., 2008; Shaw et al., 2013; Liu et al., 2019; Wolde et al., 2019; Chen et al., 2020). This shows that the influence of flowering time on FHB resistance is likely to be much more complex than previously expected.

The chromosome arms 4AL and 5BL contain two major genomic loci that showed the largest effect for DA and minor effects for HT. The 4AL QTL were found to control SEV, FDK, and DON, with the largest effect on SEV. QTL from 4A have previously been reported in durum wheat (Prat et al., 2017) and in the winter wheat cultivars Arina (Paillard et al., 2004; Lu et al., 2013; Buerstmayr and Buerstmayr, 2015) and Heyne (Zhang et al., 2012). Recently, Wu et al. (2019) identified a major QTL from the same region for type II resistance (point inoculation in the greenhouse). The previous study of FL62R1 found a minor QTL from the same region that controls resistance for point inoculation (Zhang et al., 2018). Together, results indicate that the 4AL locus plays roles for both greenhouse and field resistance. The 4AL region has been characterized intensively for structure variation with the presence of two reciprocal translocations and two inversions (Mickelson-Young et al., 1995; Miftahudin et al., 2004; Dvorak et al., 2018) between a portion of 4AL to 4DL, where a portion of 4AL corresponds to 4DL. The present study also found that this region has a paracentric inversion when comparing the genetic order of our map to the physical position of this region in the CS Ref 1.0 assembly. Wolde et al. (2019) mapped the *TB-A1*, which may have arisen from translocation of *TEOSINTE BRANCHED1* (*TB1*) from the 4DS region to the 4AL region between 629.3 and 634.8 Mb, at almost the same position as we identified the 4AL FHB QTL. *TB1* is known to play a role in controlling morphometric inflorescence traits in wheat, and we have previously identified QTL for spike density and flowering time that colocalize to the region (Zhang et al., 2018). In addition, Dixon et al. (2018) found that *TB1* interacts with *FLOWERING LOCUS T1* to regulate inflorescence architecture in bread wheat (*T*. *aestivum* L.). These findings, together with ours, indicate that *TB1* may be the candidate gene within this region for FHB resistance. We also found that FHB resistance from this region is intensely affected by environments and sites. The common parental line, FL62R1, contributed to longer DA and taller plants; in Ottawa, the locus contributed to FHB resistance while it increased FHB susceptibility at Carman. This signified that the factors responsible for local adaption also control the FHB resistance mechanism. Flowering time genes are critical to adjusting the timing of wheat flowering to its climate and can have opposite effects on fitness in different environments; thus, the locus expresses a genetic trade-off between environments (Mitchell-Olds, 2013).

The 5BL QTL region has a major effect for DA and a minor effect for height in the Stettler population, with the QTL from Stettler delaying time to anthesis. The major vernalization gene *VrnB1* is located in this region. This 5BL region conferred FHB resistance at Ottawa, having larger effects on incidence and SEV than FDK and DON. This QTL was also found in spring wheat and in Tunisian landrace durum wheat (*Qfhs.ndsu-5BL*, Ghavami et al., 2011; He et al., 2019). Currently, two major FHB resistance loci on 5AL have been reported. The first maps to the *VrnA1* region, around 584.7 Mb, and has been described in both hexaploid and durum wheat (Prat et al., 2017; Sari et al., 2018; He et al., 2019). The FHB resistance 5AL QTL from FL62R1 mapped to this region, though it only had a minor effect on flowering time and plant height. The second loci correspond to the *Q* region identified from *Triticum turgidum* ssp. *dicoccum* (Zhang et al., 2014), *Triticum macha* (Buerstmayr et al., 2011), and the hexaploid PI277012 (Chu et al., 2011), which localized around 658.1 Mb. *Q* and *VrnA1* map within 70 Mb from each other, indicating the existence of two QTL, though large phenotypic bias and relatively small population size make it possible that both QTL correspond to the same locus. Given that the QTL were identified from tetraploid to hexaploid wheat species, it is probable that the resistance gene is highly conserved from an evolutionary perspective. With this assumption, *Vrn1* and the *Q* gene, both responsible for domestication, are likely candidate genes.

We identified FHB resistance QTL for FHB syntenic region on 2AS and 2BS colocalized with the major flower control genes *Ppd-A1* and *Ppd-B1*. There was only a minor effect on DA, and the detection of the QTL was not stable across environments: the 2BS QTL was only found in the Muchmore population, and almost exclusively at Ottawa; conversely, the 2AS QTL was found in both populations, but almost exclusively at Carman. Previous studies have also identified QTL for FHB resistance in these regions (Giancaspro et al., 2016; He et al., 2019), and we previously reported a minor QTL for resistance following greenhouse point inoculation in this region (Zhang et al., 2018). *Ppd-1* has a major effect on spikelet formation, and the photoperiod insensitive allele speeds up the rate of spikelet initiation (Ochagavia et al., 2018). The *Ppd* genes are potential candidate genes for FHB resistance of both the 2AS and 2BS QTL, possibly conferring both greenhouse and field resistance through controlling the speed of flower development.

Finally, intensive research have been conducted for types I and II of FHB resistance in wheat. Despite the importance of FDK for grain quality and DON for food safety, little research was performed on FDK and DON. Recently, He et al. (2019) reported two DON QTL, 3BL, which also conferred a minor effect for FHB resistance, and 3DL, which showed no effect on field FHB resistance. Within the current study, no major QTL were identified exclusively for FDK and/or DON, but QTL with larger effect for FDK and/or DON were observed. The 2AS QTL contributed a major effect for DON and the 2BS QTL showed a major effect for FDK. The 3BS QTL had a larger effect on FDK and DON than other FHB measures, and the 5AL QTL primarily affected FDK and DON. These findings show that resistance genes for FDK and/or DON are partially shared with incidence and severity. Because of this as well as the importance of FDK and DON, there is a need to invest research on FDK and DON resistance in wheat. The QTL we identified here can be used by breeding programs as promising target QTL to develop low FDK and low DON wheat cultivars.

## Conclusion and Perspective for Future Genetic Research and FHB Resistance Breeding

The genetic architecture of high-level FHB resistance for different resistance components from FL62R1 were identified. It is concluded that FL62R1 confers Sumai 3 levels of resistance through the interaction of *Fhb1*, *Fhb5*, *RhtB1a*, and a complex flower regulatory network consisting primarily of six genomic loci controlling flower timing, including photoperiod, vernalization, and the *TEOSINTE BRANCHED1* gene. Different resistance components are partially controlled by different genetic networks and more research is needed to characterize the underlying genetic architecture. To achieve better overall FHB resistance, it is necessary to pyramid different resistant components, especially for the less well understood resistance to FDK and DON accumulation.

For future research into the genetic control of FHB resistance, there is a need to clearly characterize the important resistant loci, from the magnitude of their effects to the effect on different resistant components, especially in elite cultivar genetic backgrounds. This is especially true for the major resistance gene *Fhb1*. Given the recent cloning of two genes said to be *Fhb1* by three separate groups (Rawat et al., 2016; Li et al., 2019; Su et al., 2019), another need is to clearly characterize genes at this locus and their biological function for FHB resistance. Our findings that disease escape caused by plant height and flowering time can act as the predominant resistance mechanism and even totally mask the *Fhb1* effect emphasize the need to differentiate physiological resistance from escape. One approach is to optimize genetic mapping populations through haplotype analysis of parental lines and fixing alleles of key genes controlling flowering time and plant height. Alternatively, a subpopulation that does not segregate for these alleles can be developed by stratifying these loci from a larger mapping population through haplotype analysis and field data as practiced by Ren et al. (2019). Finally, since spray inoculation can minimize the effect of height and flower time, it should be helpful to precisely identify genomic loci contributing to physiological resistance and estimate their effects in the genetic study of FHB resistance.

From a breeding perspective, due to the complexity of the network controlling flowering time, its association with FHB resistance, and local adaption properties, it is important to completely identify the haplotypes of the major flowering time gene(s) in existing breeding programs. By fine-tuning the complex network from local adapted haplotypes (or alleles), and combining *Fhb1* and/or *Fhb5* as a base for FHB resistance with appropriate semi-dwarf alleles, desirable FHB resistance comparable to Sumai 3 levels should be achievable. The success of FL62R1 is a good example to support this breeding practice.

## Data Availability Statement

The datasets generated for this study can be found in FigShare, https://doi.org/10.6084/m9.figshare.12948263.v1.

## Author Contributions

PF conceived this study and acquired fund for this study. PF, WZ, KB, and RC designed the experiment. FJ and FE developed DH populations. HR contributed to seed increasing of DH populations. KB, PG, BP, and WZ performed the experiments. AB-B, GF, and ZR contributed to field trials and disease evaluation. WZ and KB analyzed the data and interpreted the results. WZ, KB, and PF wrote the manuscript. All authors contributed to the article and approved the submitted version.

## Conflict of Interest

The authors declare that the research was conducted in the absence of any commercial or financial relationships that could be construed as a potential conflict of interest.

## Footnotes

1 https://www.grainscanada.gc.ca/en/grain-research/export-quality/cereals/wheat/western/annual-fusarium-damage/

2 http://www.pgdc.ca/
