# Supplementary material for: Genetic Characterization of Multiple Components Contributing to Fusarium Head Blight Resistance of FL62R1, a Canadian Bread Wheat Developed Using Systemic Breeding
Source: Front Plant Sci. 2020 Oct 26;11:580833. doi: 10.3389/fpls.2020.580833 (PMC7649146; doi:10.3389/fpls.2020.580833)
Supplement: Supplementary file 2 [file Data_Sheet_1.PDF]

# *Supplementary Material*

**Figure S1**

**Article Title:** Genetic characterization of multiple components contributing to fusarium head blight resistance of FL62R1, a Canadian bread wheat developed using systemic breeding

**Journal:** Frontiers in Plant Science

Wentao Zhang, Kerry Boyle, Anita Brûlé-Babel, George Fedak, Peng Gao, Zeinab Robleh Djama, Brittany Polley, Richard Cuthbert, Harpinder Randhawa, Fengying Jiang, François Eudes, Pierre R. Fobert

**Name, affiliation, and email of corresponding author**

Pierre R. Fobert

Aquatic and Crop Resources Development,

National Research Council of Canada,

Ottawa, ON, K1A 0R6

Email: Pierre.Fobert@nrc-cnrc.gc.ca

Or

Wentao Zhang

Aquatic and Crop Resources Development,

National Research Council of Canada, Saskatoon,

SK, S7N 0W9

Email: Wentao.Zhang@nrc-cnrc.gc.ca

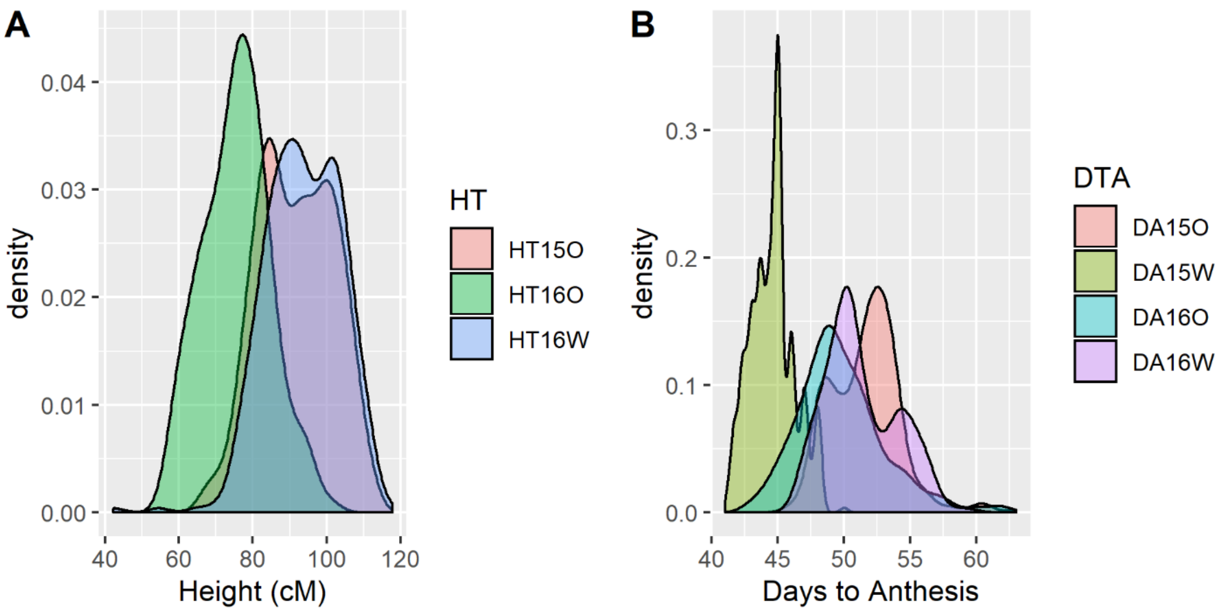


**Fig. S1** Distribution of phenotypic traits of Muchmore and Stettler populations in different field environments. (A) plant height (HT); (B) days to anthesis. HT15O, HT16O, HT16W, represent plant height measured at Ottawa, ON, in 2015, 2016 and at Carman, MB in 2016, respectively. DA15O, DA15W, DA16O and DA16W, represent days to anthesis recorded at Ottawa, ON and Carman MB, in 2015 and 2016, respectively.

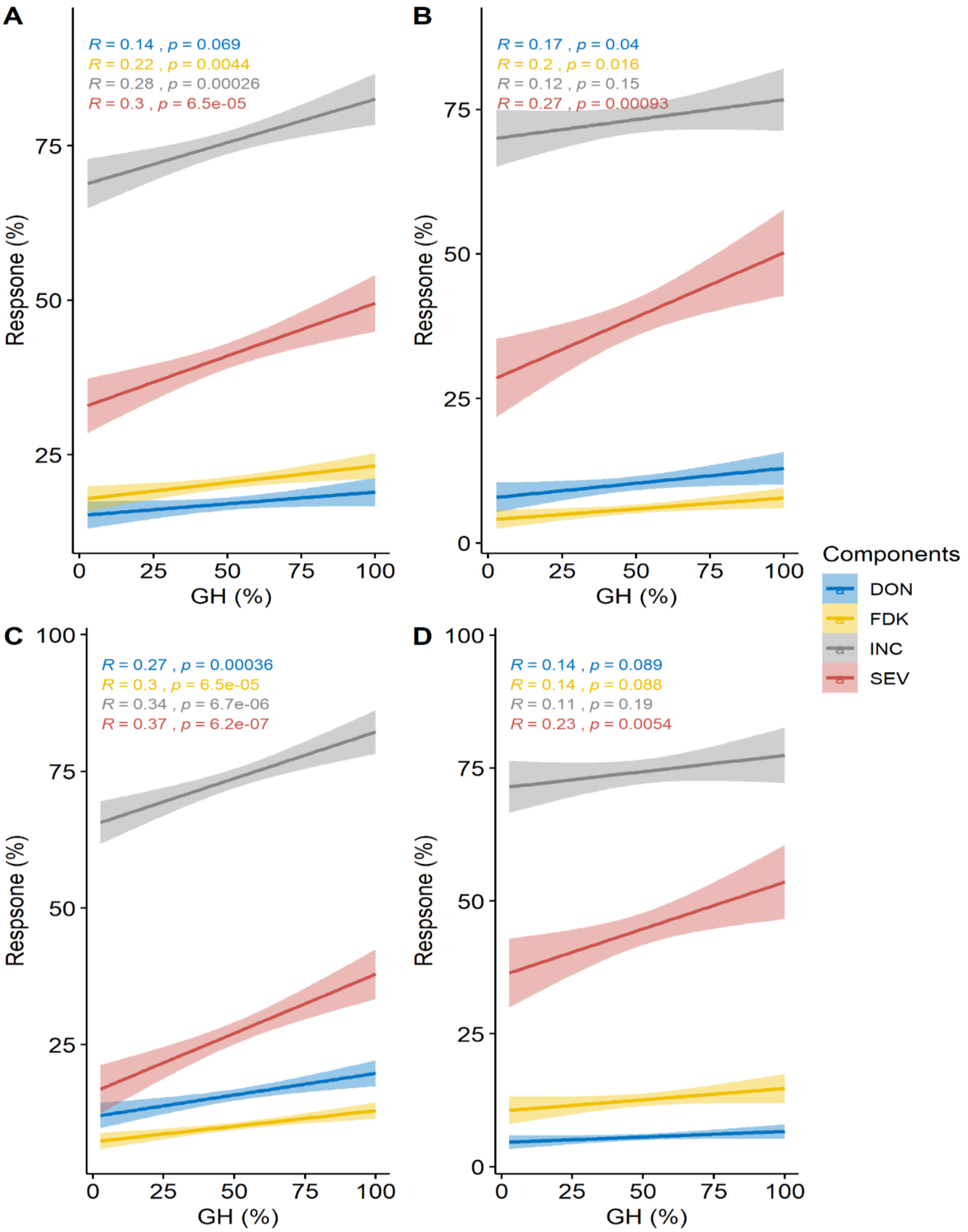


**Fig. S2** Correlation analysis between greenhouse type II (point inoculation) with field resistant components measured at: (a) Carman, MB, in 2015; (b) Ottawa, Ontario, in 2015; (c) Carman, MB, 2016; (d) Ottawa, Ontario, 2016. *INC,* incidence; *SEV*, severity; *FDK*, Fusarium damaged kernels;

*DON*, Deoxynivalenol; *HT*, plant height; *DA*, day to anthesis; *GH (%)* greenhouse FHB type II test with point inoculation from Zhang et al. (2018).
