# Supplementary material for: Genetic Characterization of Multiple Components Contributing to Fusarium Head Blight Resistance of FL62R1, a Canadian Bread Wheat Developed Using Systemic Breeding
Source: Front Plant Sci. 2020 Oct 26;11:580833. doi: 10.3389/fpls.2020.580833 (PMC7649146; doi:10.3389/fpls.2020.580833)
Supplement: Supplementary file 3 [file Data_Sheet_2.PDF]

Figure S2

Article Title: Genetic characterization of multiple components contributing to fusarium head blight resistance of FL62R1, a Canadian bread wheat developed using systemic breeding

Journal: Frontiers in Plant Science

Wentao Zhang, Kerry Boyle, Anita Brûlé-Babel, George Fedak, Peng Gao, Zeinab Robleh Djama, Brittany Polley, Richard Cuthbert, Harpinder Randhawa, Fengying Jiang, François Eudes, Pierre R. Fobert

Name, affiliation, and email of corresponding author

Pierre R. Fobert

Aquatic and Crop Resources Development,

National Research Council of Canada,

Ottawa, ON, K1A 0R6

Email: Pierre.Fobert@nrc-cnrc.gc.ca

Or

Wentao Zhang

Aquatic and Crop Resources Development,

National Research Council of Canada, Saskatoon,

SK, S7N 0W9

Email: Wentao.Zhang@nrc-cnrc.gc.ca

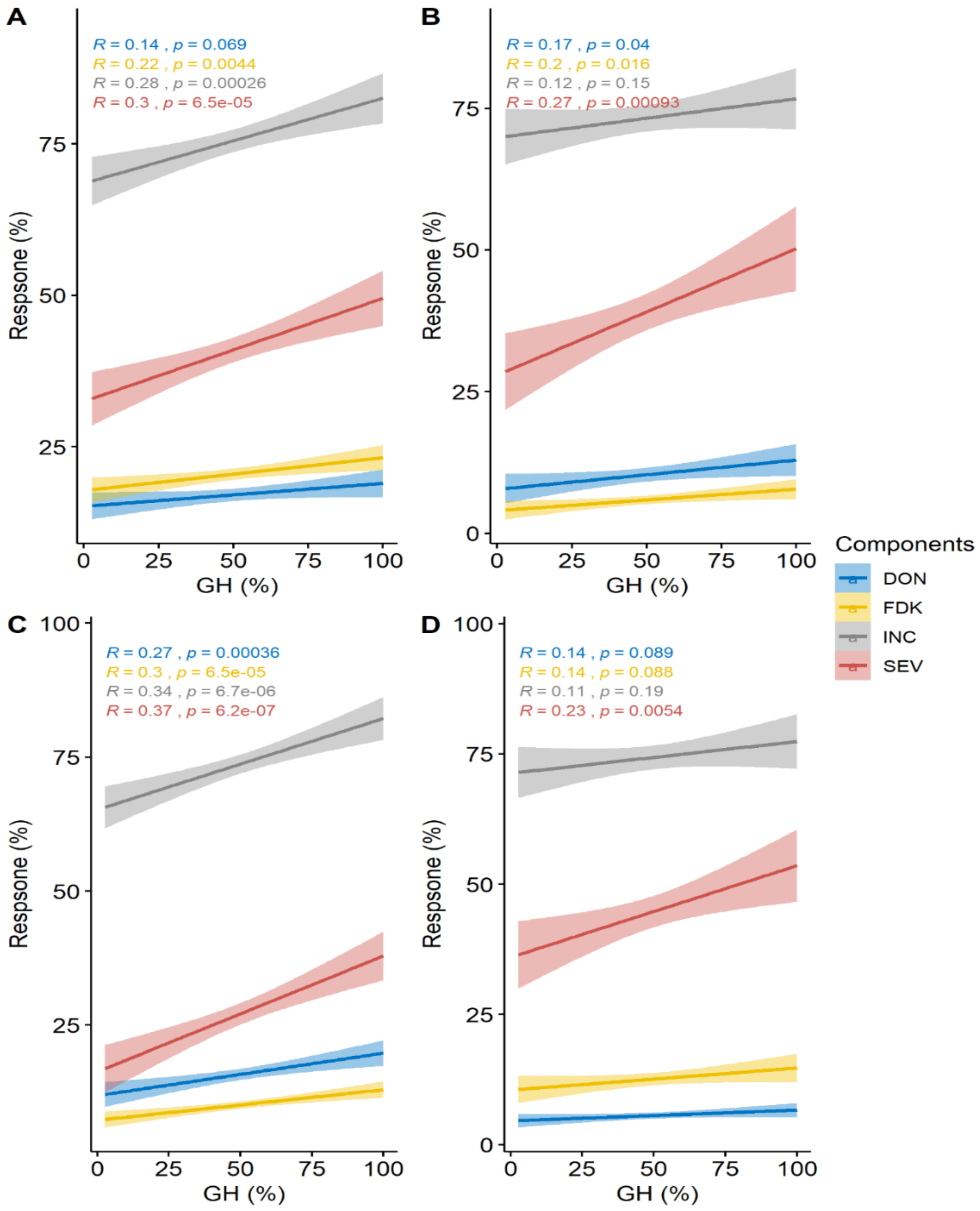

Fig. S2 Correlation analysis between greenhouse type II (point inoculation) with field resistant components measured at: (A) Carman, MB, in 2015; (B) Ottawa, Ontario, in 2015; (C) Carman, MB, 2016; (D) Ottawa, Ontario, 2016. *INC*, incidence; *SEV*, severity; *FDK*, Fusarium damaged kernels; *DON*, Deoxynivalenol; *HT*, plant height; *DA*, day to anthesis; *GH (%)* greenhouse FHB type II test with point inoculation from Zhang et al. (2018).
